# Establishing a Research Agenda for Suicide Prevention Among Veterans Experiencing Homelessness

**DOI:** 10.3389/fpsyg.2022.683147

**Published:** 2022-02-07

**Authors:** Maurand Robinson, Ryan Holliday, Lindsey L. Monteith, John R. Blosnich, Eric B. Elbogen, Lillian Gelberg, Dina Hooshyar, Shawn Liu, D. Keith McInnes, Ann Elizabeth Montgomery, Jack Tsai, Riley Grassmeyer, Lisa A. Brenner

**Affiliations:** ^1^Rocky Mountain Mental Illness Research, Education, and Clinical Center for Suicide Prevention, Aurora, CO, United States; ^2^Department of Psychiatry, University of Colorado Anschutz Medical Campus, Aurora, CO, United States; ^3^Suzanne Dworak-Peck School of Social Work, University of Southern California, Los Angeles, CA, United States; ^4^Center for Health Equity Research and Promotion, VA Pittsburgh Healthcare System, Pittsburgh, PA, United States; ^5^VA National Center on Homelessness among Veterans, Tampa, FL, United States; ^6^Department of Psychiatry and Behavioral Sciences, Duke University School of Medicine, Durham, NC, United States; ^7^Department of Family Medicine, David Geffen School of Medicine at UCLA, Los Angeles, CA, United States; ^8^Department of Health Policy and Management, UCLA Fielding School of Public Health, Los Angeles, CA, United States; ^9^VA Greater Los Angeles Healthcare System, Los Angeles, CA, United States; ^10^Department of Psychiatry, University of Texas Southwestern Medical Center, Dallas, TX, United States; ^11^VHA Homeless Programs Office, Washington, DC, United States; ^12^Center for Healthcare Organization and Implementation Research, VA Bedford Healthcare System, Bedford, MA, United States; ^13^Department of Health Law, Policy, and Management, Boston University School of Public Health, Boston, MA, United States; ^14^Department of Health Behavior, School of Public Health, University of Alabama at Birmingham, Birmingham, AL, United States; ^15^University of Texas Health Science Center at Houston, Houston, TX, United States; ^16^Department of Physical Medicine and Rehabilitation, University of Colorado Anschutz Medical Campus, Aurora, CO, United States

**Keywords:** Veterans, homelessness, housing insecurity, suicide, Delphi, suicide prevention

## Abstract

Suicide among Veterans experiencing or at risk for homelessness remains a significant public health concern. Conducting research to understand and meet the needs of this at-risk population remains challenging due to myriad factors (e.g., clinical complexity including multimorbidity, difficulty monitoring risk across systems). To address this challenge, the United States Department of Veterans Affairs (VA) convened the Health Services Research and Development (HSR&D) Suicide Prevention in Veterans Experiencing Homelessness: Research and Practice Development meeting, bringing together subject-matter experts in the fields of homelessness and suicide prevention, both from within and outside of VA. During the meeting, attendees identified 10 potential research priorities at the intersection of suicide prevention and homelessness. After the meeting, Delphi methodology was used to achieve consensus on the relative importance of the identified research domains. Through this iterative Delphi process, agreement was reached regarding the need to increase understanding of barriers and facilitators to suicide risk assessment and emergency intervention for Veterans experiencing homelessness by examining the perspectives of both Veterans and healthcare providers. Elucidating the complex relationships between risk periods, subgroups, suicide means, and drivers of suicide among Veterans experiencing homelessness was also considered a top priority. This article documents the Delphi process and provides a research agenda for researchers, funding agencies, and policymakers to prioritize the most relevant and potentially impactful research domains aimed at preventing suicide among Veterans experiencing or at risk for homelessness.

## Introduction

Veteran suicide continues to be a significant public health concern, with the rate of suicide among Veterans being 1.5 times greater than that of non-Veteran adults (Department of Veterans Affairs [VA], 2020). Among Veterans, those experiencing housing instability and homelessness have pronounced risk for suicidal self-directed violence (Hoffberg et al., 2018; Tsai and Cao, 2019; Blosnich et al., 2020b; Cusack et al., 2021). Moreover, homelessness among Veterans remains a critical focus of the VA, given that approximately 8% of all United States adults experiencing homelessness are Veterans (Department of Housing and Urban Development, 2020).

Mental health issues, including post-traumatic stress disorder and depression, within the Veteran population represent a significant public health concern (Pasinetti et al., 2021). Evidence suggests that mental health issues can be exacerbated by psychosocial factors, such as financial difficulties and housing instability (Turchi et al., 2019). For example, a recent article noted that Veterans with a history of homelessness reported significantly more severe psychiatric symptoms and greater risk for substance misuse, suicidal ideation, and suicide attempt as compared to Veterans with no history of homelessness (Holliday et al., 2021a).

Despite the well-established mental health burden and suicide risk among Veterans experiencing or at risk for homelessness, guidance regarding necessary next steps for research to prevent suicide in this population has been limited. Developing and implementing effective suicide prevention strategies that meet the needs of homeless populations is challenged by concurrent risk factors that stem from, or may be amplified by, homelessness itself. For example, individuals experiencing homelessness have high rates of early life trauma (Montgomery et al., 2013), victimization (Lee and Schreck, 2005; Hamilton et al., 2011; Pavao et al., 2013), untreated mental illness (Tsai and Rosenheck, 2015), and difficulty accessing health and social services (Omerov et al., 2020). Consequently, monitoring suicidal ideation and behavior (e.g., preparatory behavior, attempt) and expediting prevention for Veterans experiencing homelessness introduces unique challenges requiring different prevention strategies than for stably housed Veterans (Holliday et al., 2021b).

To address the complex issue of Veteran suicide concurrent with homelessness, on September 6, 2019, the VA convened the Health Services Research and Development (HSR&D) Suicide Prevention in Veterans Experiencing Homelessness: Research and Practice Development meeting in Washington, DC, United States (Holliday et al., 2021b). Meeting attendees included subject-matter experts in suicide prevention and homelessness, both from within and outside of VA, including researchers, clinicians, operational/programmatic leaders, and Veteran peer support specialists with lived experience of homelessness. The mission of the meeting was to review the state of the science regarding suicide risk associated with housing instability and homelessness within the Veteran population, and to establish a research agenda for investigators and sponsors to prioritize the most relevant and potentially impactful research domains for preventing suicide among this high-risk population.

During the meeting, attendees identified 10 potential research priorities at the intersection of suicide prevention and homelessness. After the meeting, Delphi methodology (Hsu and Sanford, 2007) was used to achieve consensus on the relative importance of the identified research domains. Delphi methodology has been used to obtain expert consensus regarding suicide prevention research methods (e.g., Nock et al., 2020) and priorities (Setkowski et al., 2020) and has been recommended as an important process for decision-making and determining collective values when clear guidance is needed (Jorm, 2015). This manuscript summarizes findings from the Delphi poll and presents a prioritized agenda for conducting research aimed at preventing suicide among Veterans experiencing or at risk for homelessness.

## Methods

In December of 2019, all 31 meeting attendees were invited by email to participate in the online Delphi poll survey. Based on prior research (Hsu and Sanford, 2007), it was pre-determined that up to three survey rounds would be utilized as necessary to achieve consensus for all research domains. All survey iterations were developed and administered online in REDCap. Responses remained confidential throughout the process. Consensus criteria was pre-established as ≥70% of respondents choosing the most frequently selected rating or the rating one point below or above the most frequently selected rating (i.e., the mode ± 1).

### Round One

Meeting attendees received an email inviting them to participate in the survey, including a link to Round One of the survey and the response deadline (3 weeks). The invitation email included that survey responses would only be shared in an aggregate and/or de-identified manner. A reminder email was sent before the response deadline. Respondents were asked to provide their first and last names, so that unique survey links could be generated for future survey rounds, as well as their current affiliation and role in the fields of suicide prevention and/or homelessness.

Respondents were presented with the 10 research domains identified during the meeting and asked to rate the importance of each domain using a nine-point Likert scale, ranging from one (not at all important) to nine (extremely important). Respondents did not receive any additional descriptors of the numbers on the scale. After selecting their rating for each domain, respondents had the option to provide justification for their selection using free text response. For example, respondents could justify the score they selected by emphasizing how this domain would further enhance research and/or clinical care related to homeless Veteran suicide prevention. Respondents were also presented with four supplementary questions: (1) Are there any domains missing from the list?; (2) Should any of the listed domains be removed from the list?; (3) Should two or more of the listed domains be collapsed into one domain?; and, (4) Should any of the listed domains be re-worded? Individuals who answered yes to any of these questions were asked to specify which domain(s) and to justify their response. Only individuals who responded to Round One of the survey were invited to participate in Rounds Two and Three.

### Rounds Two and Three

An email was sent to each Round One respondent inviting them to participate in Round Two of the survey and containing a unique survey link and the response deadline (3 weeks). A reminder email was sent before the response deadline. Research domains for which consensus criteria were met during Round One were removed from Round Two of the survey, but the remaining research domains were otherwise presented in the same order as in Round One. For each remaining research domain, respondents were presented with their individual Round One score, the Round One mean score, and the Round One score range. Respondents were also presented with anonymized score justifications provided by Round One respondents. After being presented with this information, respondents were asked to re-rate the importance of each domain using the same nine-point Likert scale used in Round One. Respondents could choose the same score they had selected in Round One or a different score. Round Two procedures were replicated during Round Three.

## Results

### Respondents

Of the 31 meeting attendees invited to participate, 11 (35.5%) completed Round One of the Delphi survey, consistent with response rates seen for other Delphi processes (Hsu and Sanford, 2007). All respondents reported being affiliated with the VA, with the majority indicating their current role as “VA Research.” All 11 (100%) Round One respondents also completed Rounds Two and Three of the survey.

### Delphi Process

During Round One, consensus criteria were met for five of the ten research domains. A majority of respondents answered “no” to the supplementary questions, so no research domains were added, removed, collapsed, or reworded. During Round Two, consensus criteria were met for three of the five remaining research domains. Finally, in Round Three, consensus criteria were met for one of the two remaining research domains. The nine research domains for which consensus was achieved are ordered by mean importance score in Table 1, with a higher mean score representing the domain being rated as more important. Consensus criteria were not met after three rounds of the Delphi process for one domain (i.e., “Examine novel approaches that focus on destigmatizing mental health treatment and addressing loneliness among Veterans experiencing homelessness, including use of peer-support specialists, adjunctive VA programming (e.g., Chaplains), medical-legal partnerships, and current case managers.”). As outlined in the Methods section, it had been pre-determined that up to three survey rounds would be utilized to achieve consensus, based on prior research demonstrating that if consensus is not reached within three rounds consensus is unlikely to be reached with additional rounds (see Hsu and Sanford, 2007). For this reason, subsequent survey rounds were not conducted.

**TABLE 1:** Agenda for research on suicide prevention among veterans experiencing homelessness.

Research priority		Mean ± SD*
1	Examine barriers and facilitators to suicide risk assessment and emergency intervention in the community with homeless Veterans by obtaining both provider and Veteran perspectives.	8.18 ± 0.98
2	Identify the complex relationships between periods of risk, subgroups at elevated risk, suicide means, and clinical and non-clinical drivers (e.g., social determinants of health) of suicide risk among homeless Veterans. Delineate how these factors differ based on factors such as gender, race/ethnicity, rurality, and age.	8.09 ± 1.58
3	Obtain further understanding of the specific needs of homeless Veterans experiencing elevated risk for suicide to determine how current evidence-based treatment (e.g., Safety Planning, Cognitive Behavioral Therapy for Suicide Prevention) can be tailored to the needs of this population.	7.91 ± 1.58
4	Identify upstream drivers of suicidal self-directed violence among homeless Veterans through novel methodological and analytic approaches, such as merging VA and non-VA datasets, mixed-method approaches, and advanced modeling (e.g., structural equation modeling, network analysis).	7.73 ± 1.01
5	Understand how VA providers evaluate and respond to suicide risk with homeless Veterans.	7.45 ± 0.82
6	Conduct a prospective cohort study and a retrospective cohort study of homeless Veterans to determine predictors of suicidal self-directed violence in this population.	7.36 ± 1.12
7	Understand the life course trajectory of homeless Veterans who have been suicidal to understand how cumulative adverse events contribute to negative outcomes.	6.64 ± 1.29
8	Examine the role of data regarding finances (e.g., Veterans Benefits Association benefits) in enhancing understanding of risk for suicide among homeless Veterans.	6.64 ± 2.42
9	Identify biological markers of suicide among homeless Veterans (e.g., Million Veteran Program).	2.91 ± 1.70

*All ratings were made on a Likert scale ranging from one (“Not at all Important”) to nine (“Extremely Important”).

## Discussion

Through an iterative Delphi process, subject matter experts came to consensus regarding the relative importance of nine of the ten research domains identified at the VA HSR&D Suicide Prevention in Veterans Experiencing Homelessness: Research and Practice Development meeting. In addition, respondents rated several domains as particularly important for preventing suicide among Veterans experiencing or at risk for homelessness.

Agreement was reached regarding the need to increase understanding of barriers and facilitators to suicide risk assessment and emergency intervention for Veterans experiencing homelessness by examining the perspectives of both Veterans and healthcare providers. Elucidating relationships between risk periods, subgroups, suicide means, and drivers of suicide was also considered a top priority. Indeed, discussion of this domain at the meeting itself has already prompted new research in this area, including a recent investigation of differences in methods of suicide based on housing stability (Blosnich et al., 2020a). Consensus was also reached regarding the need to increase understanding of the specific needs of Veterans experiencing homelessness to determine how current evidence-based treatments (e.g., Safety Planning, Cognitive Behavioral Therapy for Suicide Prevention) can be tailored to the needs of this population.

Domains involving the use of specific research methodologies to identify upstream drivers of suicide, including prospective and retrospective cohort studies, analysis of financial data (e.g., Veterans Benefits Administration data), and taking a life course perspective, were also considered important. The infrequent use of these methodological approaches in research on suicide risk and prevention among Veterans experiencing homelessness has been noted previously (Holliday et al., 2021b). Such approaches could optimize existing data, while also potentially addressing limitations of prior methods (e.g., sole focus on Veterans Health Administration data; assessment of medical, but not social service records), thus enhancing understanding and provision of health services for this at-risk population.

In contrast, identifying biological markers of suicide in Veterans experiencing homelessness was considered a relatively lower priority. Given the domains with the highest ratings, respondents may have prioritized domains that identified immediate methods of enhancing access to, and benefit from, acute and ongoing clinical care. Conversely, while identifying biological markers of suicide may have potential benefit, such an approach may take longer to impact suicide rates and thus have been perceived as a lower priority.

Research domains with the greatest potential to inform and advance suicide prevention efforts among Veterans experiencing or at risk for homelessness were elucidated *via* this Delphi process; however, continuing conversations must address how to mobilize these topics into impactful research. For example, VA research centers have utilized Veteran engagement groups to promote health services research specific to the unique needs of Veterans (Wendleton et al., 2019). Both sponsors and researchers should create opportunities for community-based participatory research that engages Veterans with lived experience of homelessness to inform research projects, generate ideas for recruitment and retention, and explore the use of technology to expedite participation.

While these findings provide new direction and priorities for future research aimed at preventing suicide among Veterans experiencing or at risk for homelessness, specific limitations should be considered. In particular, only a portion (35.5%) of those invited to participate responded to the survey, the majority of whom were affiliated with the VA and described their role as primarily involving research. Although low response rates are a common issue when undertaking Delphi polls (Hsu and Sanford, 2007), this limitation suggests that our findings may largely reflect VA researchers’ perspectives, and may not be germane to other key stakeholders, such as policymakers, non-VA researchers, and/or Veterans themselves. It is important to note, however, that a larger, more diverse group (inclusive of non-VA researchers and Veterans) identified the domains utilized in the Delphi process, suggesting that the domains are potentially more inclusive of a broader range of perspectives than the Delphi sample. Nonetheless, future research would benefit from examining if differences in ratings of priorities emerge based on affiliation and/or background.

Despite these limitations, our findings underscore that there is consensus among VA suicide prevention and homelessness researchers regarding several important research priorities necessary to prevent suicide among Veterans experiencing or at risk for homelessness. We urge researchers and policymakers alike to attend to these findings and are hopeful for next steps in preventing suicidal self-directed violence among this at-risk population of Veterans.

## Data Availability Statement

The raw data supporting the conclusions of this article will be made available by the authors, without undue reservation.

## Ethics Statement

Ethical review and approval was not required for this study on human participants in accordance with the local legislation and institutional requirements. Written informed consent for participation was not required for this study in accordance with the national legislation and the institutional requirements.

## Author Contributions

MR led the writing of this manuscript as well as the Delphi process. MR was assisted by RH, LM, LB, and RG. JB, EE, LG, DH, SL, DM, AM, and JT facilitated in editing the manuscript. All authors contributed to the article and approved the submitted version.

## Conflict of Interest

The authors declare that the research was conducted in the absence of any commercial or financial relationships that could be construed as a potential conflict of interest.

## Publisher’s Note

All claims expressed in this article are solely those of the authors and do not necessarily represent those of their affiliated organizations, or those of the publisher, the editors and the reviewers. Any product that may be evaluated in this article, or claim that may be made by its manufacturer, is not guaranteed or endorsed by the publisher.
